# Metals and cholesterol: two sides of the same coin in Alzheimer’s disease pathology

**DOI:** 10.3389/fnagi.2014.00091

**Published:** 2014-05-15

**Authors:** Bruce X. Wong, Ya Hui Hung, Ashley I. Bush, James A. Duce

**Affiliations:** ^1^Oxidation Biology Unit, The Florey Institute of Neuroscience and Mental Health, The University of MelbourneParkville, VIC, Australia; ^2^School of Molecular and Cellular Biology, Faculty of Biological Sciences, University of LeedsLeeds, North Yorkshire, UK

**Keywords:** Alzheimer’s disease, amyloid precursor protein, Aβ, cholesterol, metals, iron, copper, zinc

## Abstract

Alzheimer’s disease (AD) is a multifactorial neurodegenerative disease. It begins years prior to the onset of clinical symptoms, such as memory loss and cognitive decline. Pathological hallmarks of AD include the accumulation of β-amyloid in plaques and hyperphosphorylated tau in neurofibrillary tangles. Copper, iron, and zinc are abnormally accumulated and distributed in the aging brain. These metal ions can adversely contribute to the progression of AD. Dysregulation of cholesterol metabolism has also been implicated in the development of AD pathology. To date, large bodies of research have been carried out independently to elucidate the role of metals or cholesterol on AD pathology. Interestingly, metals and cholesterol affect parallel molecular and biochemical pathways involved in AD pathology. The possible links between metal dyshomeostasis and altered brain cholesterol metabolism in AD are reviewed.

## INTRODUCTION

Alzheimer’s disease (AD) is a multifactorial neurodegenerative disease characterized by pathological hallmarks of extracellular β-amyloid (Aβ) plaques (Glenner and Wong, 1984a,b; Masters et al., 1985) and intracellular neurofibrillary tangles (Delacourte and Defossez, 1986; Kosik et al., 1986; Lee et al., 1991) in the brain. The rate of AD progression is variable, but on average, patients may live up to 10 years after diagnosis (Whitehouse, 1997). Approximately 8–10% of the population over the age of 65 have AD, and its prevalence doubles every 5 years thereafter (Cummings, 2004; Bertram and Tanzi, 2005). These data, coupled with ever increasing life expectancy, marks AD as one of the most significant health and socio-economic problems, particularly in industrialized nations.

As with most diseases, genetic and environmental factors can contribute to its development. AD can be broadly characterized as either familial or sporadic. Early-onset familial AD (FAD) are caused by mutations within three genes, which encode the amyloid precursor protein (*APP*) and presenilins 1 and 2 (*PSEN1* and *PSEN2*; Holmes, 2002; Tanzi and Bertram, 2005; Bertram et al., 2007). These mutations are autosomal dominant, and symptoms of AD manifest prior to 65 years of age. FAD accounts for less than 5% of AD cases (Janssen et al., 2003; Raux et al., 2005). The disease etiology for late-onset sporadic AD is complex and multifactorial, which may involve age-related alterations in metabolism, repair mechanisms, immune response, and environmental factors such as life style, prior brain trauma, and oxidative stress (Muller-Spahn and Hock, 1999; Chen et al., 2009). Genome-wide association studies (GWAS) have identified candidate genes that significantly increase the risk of late-onset AD. By far, the strongest risk factor found is the ε4 allele of the apolipoprotein E (*APOE*) gene (Farrer et al., 1997). Possessing just a single ε4 allele increases the risk of developing AD by two- to fivefold, while having two alleles increases the risk to more than fivefold (Poirier et al., 1993; Strittmatter et al., 1993; Holmes, 2002; Poirier, 2003; Bertram et al., 2007; Coon et al., 2007).

Neuritic plaques are multi-cellular lesions containing Aβ peptides (especially the neurotoxic Aβ_42_ species), reactive astrocytes, activated microglia, and dystrophic neurites (Maulik et al., 2013). Aβ peptide is produced by the proteolytic cleavage of APP by β- and γ-secretases (see APP Processing and Aβ Generation). Interestingly, these plaques also have an enrichment of cholesterol (Panchal et al., 2010) and metals such as copper, iron, and zinc (Goodman, 1953; Connor et al., 1992; Bush et al., 1994c; Lovell et al., 1998; Suh et al., 2000; Collingwood et al., 2005; Stoltenberg et al., 2005; Miller et al., 2006; Baltes et al., 2011), which indicate a failure of cholesterol and metal regulatory systems in the brain. While the underlying etiology of AD is yet to be clearly established, mounting evidence derived from epidemiological, clinical and biochemical studies have independently implicated roles for metals and cholesterol in the pathogenesis of AD. This review presents an overview of the roles of metals and cholesterol in APP/Aβ metabolism and their relationship in the development of AD.

## APP PROCESSING AND Aβ GENERATION

**FIGURE 1:**
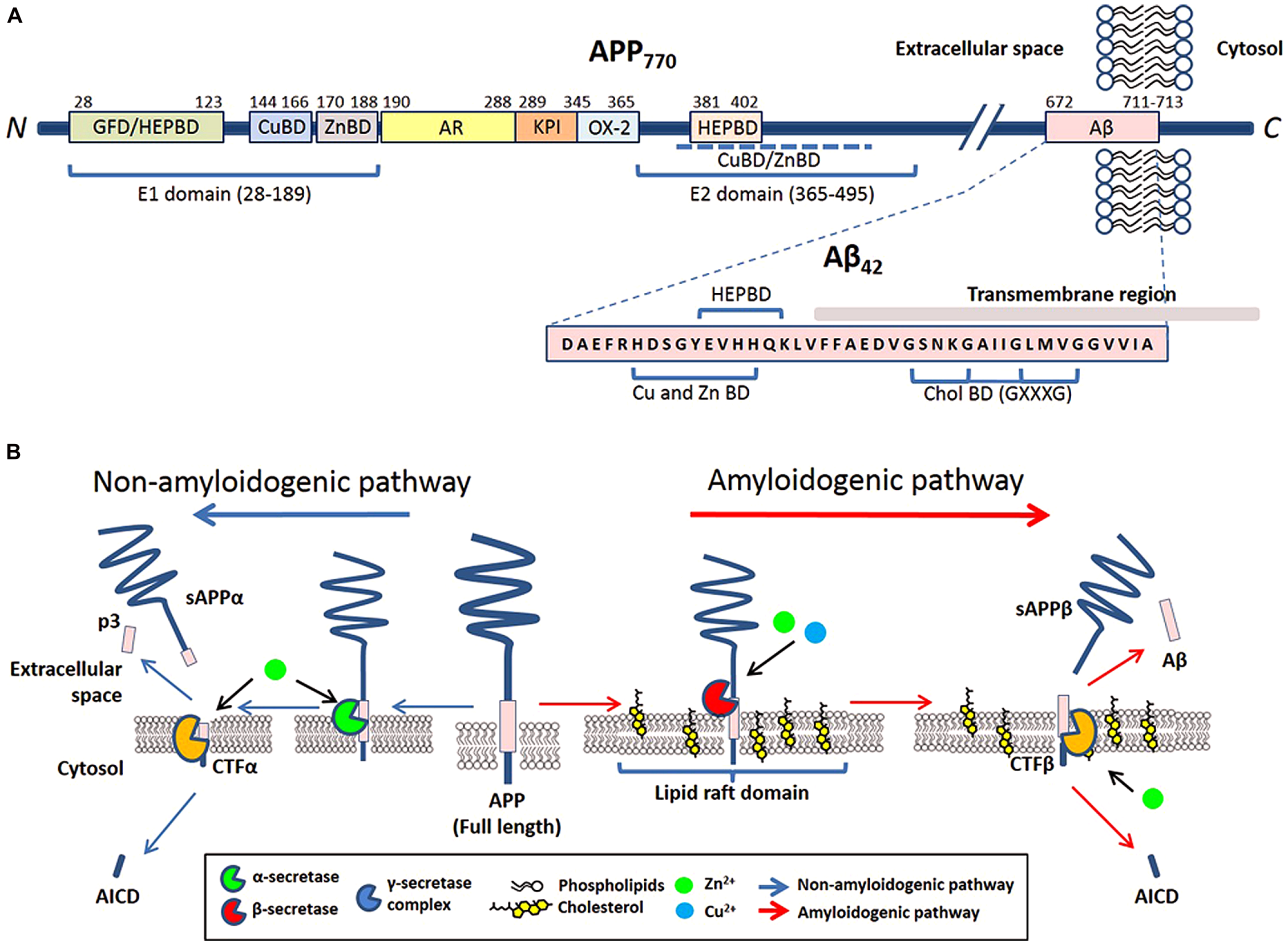
**The involvement of metals and cholesterol in post-translational modification of APP. (A)** Schematic of reported metal and cholesterol binding domains in APP_770_ and Aβ in relation to other recognized motifs. APP_770_ is the longest isoform of APP with the APP_751_ isoform lacking the OX-2 domain and the neuron prevalent isoform APP_695_ lacking both OX-2 and Kunitz-type protease inhibitor (KPI). Within the extracellular presented ectodomain of APP, the E1 region at the N-terminal contains a copper binding domain (CuBD) and zinc binding domain (ZnBD) that is C-terminally orientated compared to the growth factor domain (GFD) which incorporates a heparin binding domain (HEPBD). The E1 domain is followed by the acidic region (AR), KPI and OX-2 before the E2 domain of APP, containing a HEPBD and CuBD/ZnBD that is yet to be exactly mapped (Dahms et al., 2012). The E2 domain is followed by the Aβ peptide that is partially embedded into the transmembrane region. Aβ also has a recognized CuBD/ZnBD as well as a Cholesterol binding region (CholBD) that incorporates the GXXXG motifs. **(B)** Proteolytic processing of APP predominantly follows two pathways that are initiated by separate secretases. The non-amyloidogenic pathway (*blue arrows*) initiates with the cleavage of full-length APP by α-secretase within the Aβ sequence. Following further cleavage by the γ-secretase complex, this pathway results in the generation of soluble N-terminal APP fragment (sAPPα) and C-terminal fragments (p3 and AICD). The alternative amyloidogenic pathway (*red arrows*) involves sequential cleavage of APP by β-secretase followed by the γ-secretase complex, which results in the liberation of a soluble N-terminal sAPPβ fragment, Aβ peptide, and AICD. Copper and zinc affect the processing of APP and Aβ generation on neuronal membranes through their direct influence on the enzymatic activity of β-, α-, and γ-secretases. The influence of cholesterol is through its requirement in lipid raft domains, the location for amyloidogenic processing of APP.

Amyloid precursor protein is a type I trans-membrane protein that can be post-translationally modified by *N*- and *O*-glycosylation, tyrosine sulfation, and phosphorylation (Weidemann et al., 1989; Walter et al., 2000). Full-length APP is sequentially processed via two pathways: the non-amyloidogenic and amyloidogenic. The cleavage by α- or β-secretases at the N-terminus of the Aβ domain generates soluble APP derivatives: sAPPα and sAPPβ, respectively, leaving behind membrane tethered C-terminal fragments (CTFα and CTFβ, respectively). Subsequent cleavage of these CTFs by the γ-secretase generates either p3 (from CTFα) or Aβ (from CTFβ), and liberation of the APP intracellular domain (AICD; reviewed in Zheng and Koo, 2011; **Figure 1B**).

The trans-membrane aspartyl protease β-site APP cleaving enzyme 1 (BACE1) is the major β-secretase in neurons (Sinha et al., 1999). This is the rate-limiting enzyme involved in the generation of Aβ (Vassar et al., 1999; Yan et al., 1999). In contrast, α-secretase cleavage of APP can be stimulated by a disintegrin and metalloproteinase (ADAM) family of proteases (reviewed in Lichtenthaler, 2011) and a number of other molecules (e.g., phorbol ester) or via protein kinase C activation, in which case the cleavage is regulated by tumor necrosis factor α-converting enzyme (TACE; Buxbaum et al., 1998; Blacker et al., 2002). Studies have indicated that in neurons, α-secretase activity is likely to be primarily mediated by ADAM10 (Kuhn et al., 2010). The mature γ-secretase is a polytopic complex consisting of four individual components: presenilin (PS); nicastrin (Nct); anterior pharynx defective 1 (Aph1); and presenilin enhancer 2 (Pen-2; reviewed in Edbauer et al., 2003; Iwatsubo, 2004). Presenilin, an aspartyl protease, is the main catalytic unit of the complex. Aβ of varying length is the result of hierarchical and site-specific cleavage of APP by β- and γ-secretase (**Figure 1B**). Other than APP, all three APP-cleaving secretases can digest other biological substrates required for multiple biological functions such as regulation of development, differentiation, and proliferation.

## METAL AND CHOLESTEROL MODULATION OF APP AND Aβ METABOLISM

The dynamics of biological metal ions (e.g., copper, zinc, and iron) is critical for many physiological functions. Metal ions are key components in many enzymatic functions, which include catalysis, structural stability, transportation of oxygen, and cellular signaling. The passive flux of metals between the circulation and the brain is tightly regulated by the blood–brain barrier (BBB; Duce and Bush, 2010). The impact of metals on the brain causing neurodegeneration may be caused by increased toxic exposure, as well as a breakdown in the mechanisms that compartmentalize and regulate metal homeostasis.

The brain is the most cholesterol-rich organ in the body. Functionally, cholesterol plays a critical role in neuronal development and maintenance of synaptic plasticity. As a component of the plasma membrane, it regulates ion homeostasis, endocytosis, and intracellular signaling pathways. It also serves as a precursor for the production of steroid hormones, vitamin D, and oxysterols. Like metals, experimental work has shown compartmentalization between levels of cholesterol in the serum and brain that is regulated by BBB (Hung et al., 2013). Substantial evidence correlates cholesterol homeostasis dysregulation with AD. In cell culture systems, production of Aβ is linked to cholesterol levels. However, the exact influence of cholesterol in Aβ generation is still unclear.

### METALS AND APP

The APP sequence contains putative binding sites for copper (Hesse et al., 1994; Atwood et al., 2000; Simons et al., 2002; Barnham et al., 2003; Valensin et al., 2004) and zinc (Bush et al., 1993, 1994a,b,c). Copper binds to APP between residues 142 and 166 (White et al., 1999a; Barnham et al., 2003), a site where it can also catalytically reduce copper (Multhaup et al., 1996). Recently, two copper binding residues at histidine 149 and 151 have been identified as crucial for APP metabolism, protein folding and stability (Spoerri et al., 2012). The Aβ segment of APP is another region that directly interacts with copper and is explained in more detail in Section “Metal Modulation of Aβ Generation, Aggregation, and Cell Toxicity.” The N-terminal copper binding domain of APP has been found to play crucial roles in homodimerization (Hesse et al., 1994; Kaden et al., 2008), and an elevation in copper levels increases APP homodimerization (Noda et al., 2013). Zinc binds to a conserved region of amino acids between position 170 and 188 of APP (Bush et al., 1993, 1994a). The coordination binding involves two key cysteines at positions 186 and 187, as well as other potential ligands (e.g., C174, M170, D177, and E184). Similar to copper, the binding of zinc may also play an important functional role in homodimerization of APP (Scheuermann et al., 2001; Ciuculescu et al., 2005; **Figure 1A**).

A number of *in vivo* and *in vitro* studies highlight the reciprocal regulation between APP and metal ions. The regulation of *APP* gene expression is linked to altered cellular copper levels. Studies in the *in vitro* cell culture show that copper depletion by overexpressing copper transporter ATP7A result in down-regulation of *APP* gene expression and APP protein level; conversely, *APP* gene expression level is up-regulated under conditions of copper overload due to ATP7A-deficiency (Armendariz et al., 2004; Bellingham et al., 2004b). On the other hand, copper concentration is increased in brain and liver tissue as well as primary neuronal and skin fibroblast cells from APP and amyloid precursor-like protein 2 (APLP2) knockdown mice (White et al., 1999b; Bellingham et al., 2004a; Hung et al., 2009; Acevedo et al., 2011). The difference in copper level is even more pronounced in aged mice (Needham et al., 2014). In contrast, APP over-expressing transgenic mice have decreased copper in the brain (Maynard et al., 2002; Bayer et al., 2003; Phinney et al., 2003). Copper treatment stimulates the movement of APP from the *trans*-Golgi network to the plasma membrane and attenuates internalization of APP to BACE1-rich endosomes. However, copper treatment does not result in any detectable change in APP processing (Hung et al., 2009; Acevedo et al., 2011). In humans, low copper diet is associated with a significant decrease in APP expression in platelets from healthy postmenopausal women (Davis et al., 2000).

Iron regulates APP translation, which involves an iron response element (IRE) RNA stem loop in its 5′-untranslated region (UTR). The APP IRE is homologous with the canonical IRE RNA stem-loop that binds iron regulatory proteins (IRP1 and IRP2) to control intracellular iron homoeostasis by modulating ferritin mRNA translation and transferrin receptor mRNA stability (Rogers et al., 2002). IRP1, but not IRP2, selectively binds to the APP IRE in human neural cells (Cho et al., 2010). Intracellular metal chelation selectively down-regulates APP 5′-UTR translation, which is reversed by cytoplasmic labile iron (Venti et al., 2004). The regulation of APP by iron through the 5′-UTR indicates that iron has a role in APP metabolism.

In the brain, ferroportin (Fpn) is required for excess iron to exit the cell (Donovan et al., 2005; Ganz, 2005). Fpn channels transport iron through the plasma membrane where it is required to be converted to its ferric form before being released and loaded onto transferrin, the extracellular iron-transporting protein that transfers iron between cells (Swaiman and Machen, 1984). APP may play a role in the iron export mechanism of cells through the stabilization of Fpn (Duce et al., 2010). APP knockout mice exposed to dietary iron results in ferrous iron accumulation and oxidative stress in cortical neurons. Ablation of APP in HEK293T cells and primary neurons negates iron export, which can be restored by the addition of exogenous APP (Duce et al., 2010). This iron-export capability of APP requires tau to traffic endogenous APP to the cell surface (Lei et al., 2012).

### CHOLESTEROL AND APP

Cholesterol is not symmetrically distributed laterally and between the two leaflets of the lipid membrane bilayer. The significance of this asymmetry is not yet known, although cholesterol has been implicated in cell membrane fluidity, integrity, and function (Wood et al., 1999; Hayashi et al., 2002). Patches of the membrane highly enriched with cholesterol and sphingolipid are termed lipid rafts (also known as detergent-resistant microdomains). Cholesterol provides structural stability in rafts by serving as a molecular spacer, filling in voids between raft proteins and other raft lipids such as sphingolipids and gangliosides (Xu and London, 2000; Ramstedt and Slotte, 2006). Therefore, modulation of cholesterol can result in dissociation, dysregulation, and/or inactivation of raft proteins. Indeed, APP processing and activity is influenced by its membrane domain localization.

Binding of cholesterol to APP occurs in the trans-membrane carboxyl-terminal region between amino acids 672 and 770 (or CTFβ) through interactions with membrane-buried GXXXG motifs (G, glycine; X, any amino acid; Barrett et al., 2012; **Figure 1A**). The GXXXG motif is involved with APP homodimerization (Kim et al., 2005; Munter et al., 2007; Kienlen-Campard et al., 2008; Miyashita et al., 2009; Sato et al., 2009). Competitive studies of C99 with cholesterol suggest that complexing of cholesterol:C99 at a 1:1 ratio is preferred over C99 homodimers under most physiological conditions (Song et al., 2013). The binding of cholesterol directly to APP and CTFβ may promote amyloidogenic processing by increasing the localization of APP/CTFβ to cholesterol-rich membrane domains and organelles, where γ- and β-secretases preferentially reside (Beel et al., 2010).

In cultured rat neuronal cells, up-regulation of *APP* gene expression reduces cholesterol biosynthesis while down-regulation of *APP* gene expression has the opposite effect (Pierrot et al., 2013). Membrane cholesterol content, however, is not affected. Sterol receptor element binding protein (SREBP) and rate limiting enzyme HMG-CoA reductase (HMGCR) control biosynthesis of cholesterol. The site-2 zinc metalloprotease (S2P) cleaves SREBP at Site-2 within the membrane-spanning domain (Brown and Goldstein, 1999). Interaction of APP with SREBP1 prevents S2P-mediated processing of mSREBP1 nuclear translation of its target genes including HMGCR (Pierrot et al., 2013). Interestingly, the APP/Aβ GXXXG motif is critical in the regulation of HMGCR. In contrast to neuronal cells, APP interaction with SREBP1 and resulting cholesterol biosynthesis is not detectable in astrocytes (Pierrot et al., 2013). APP expression associated reduction of cholesterol and oxysterol production is mediated via down-regulation of both HMGCR and 24-hydroxylase [required to convert cholesterol to 24S-hydroxycholesterol (24OHC)] activities, respectively. Since membrane cholesterol remains the same, it is suggested that APP controls cholesterol turnover (Pierrot et al., 2013).

### METAL MODULATION OF APP PROCESSING ENZYMES

Metals can indirectly affect Aβ generation by altering secretase-dependent processing of APP. To date, all three secretases involved in APP cleavage are known to have interactions with different metal species. The α-secretase TACE contains a zinc ion in its catalytic domain (Cross et al., 2002). TACE enzymatic activity is controlled by a “cysteine-switch” motif mediated by an intramolecular bond between cysteine and a zinc atom in its catalytic site. Subsequently, it has been shown that other regions of the TACE prodomain are able to circumvent the “cysteine-switch” and inhibit enzymatic activity (Buckley et al., 2005). Correspondingly, the metalloprotease ADAM10 can be inhibited by its dominant-negative form that has a point mutation in its zinc-binding site (Lammich et al., 1999).

The major β-secretase BACE1, binds copper in its C-terminal domain, the same region that interacts with domain I of copper chaperone for superoxide dismutase-1 (CCS; Angeletti et al., 2005). The expression of BACE1 reduces superoxidase 1 (SOD1) activity. In contrast, in cells overexpressing both BACE1 and CCS, SOD1 activity is restored by CCS (Angeletti et al., 2005). An interaction between BACE1 and CCS has been demonstrated by co-immunoprecipitation from brain homogenates and their co-transport through the axon (Angeletti et al., 2005).

Presenilin, the active subunit of the γ-secretase, is also sensitive to metal levels. Neonatal cortical cultures exposed to zinc increases C-terminal fragmentation of PS1 by enhancing synthesis of the protein (Park et al., 2001). However, zinc induces oligomerization of an APP γ-secretase substrate and inhibits its processing, which supports a role for zinc dysregulation in Aβ processing (Hoke et al., 2005; Greenough et al., 2011).

Taken together, these results suggest a direct influence of metals on secretase enzymatic activity to process APP and therefore may have detrimental implications in AD pathology when metal homeostasis is altered.

### CHOLESTEROL MODULATION OF APP PROCESSING

Previous studies show that full-length APP, Aβ, APP-CTFs, and PS1 are associated with lipid rafts (Lee et al., 1998; Simons et al., 2001; Hur et al., 2008). Studies with cultured cells demonstrate cholesterol depletion by β-cyclodextrin extraction or inhibition of cholesterol biosynthesis by statins (Simons et al., 1998; Wahrle et al., 2002), result in decreased Aβ production. Conversely, increasing cellular cholesterol levels enhance Aβ production and reduce α-secretase cleavage of APP (Bodovitz and Klein, 1996; Frears et al., 1999).

Since APP, β- and γ-secretases are associated with lipid raft domains, it is not surprising that altered cellular cholesterol content affects Aβ generation, aggregation, and clearance. The presence of lipid raft domains has been found in plasma membranes and endosomes. More recently, a study uncovered lipid raft-like domains in mitochondria-associated endoplasmic reticulum (ER) membranes (MAMs), a sub-compartment of the ER connected to mitochondria (Area-Gomez et al., 2012). Lipid rafts are sensitive to altered cholesterol metabolism, and cholesterol depletion results in lipid raft destabilization (Eckert et al., 2010). As previously mentioned, cholesterol enriched in lipid rafts can influence dynamics of proteins within these rafts. Altered cholesterol levels affect lipid raft localization of APP and its derivatives together with secretases required for APP processing. Biochemical isolation of lipid rafts indicates that BACE1 and γ-secretase protein are localized within these lipid domains (Wahrle et al., 2002; Vetrivel et al., 2004; Kalvodova et al., 2005; Osenkowski et al., 2008), while the α-secretase ADAM10 is predominantly localized outside the lipid rafts (Kojro et al., 2001). Consistent with other lipid raft domains, MAMs have a high concentration of APP, PS1, and PS2 (catalytic subunits of γ-secretase) and γ-secretase activity. APP is believed to exist in either pool within plasma membranes (Ehehalt et al., 2003). Experimental evidence suggests that amyloidogenic processing of APP occurs in lipid rafts while the non-amyloidogenic processing occurs mainly in the non-raft regions. If this is the case, then cholesterol levels contribute to regulation of APP processing through these two pathways. The non-amyloidogenic pathway predominates, because only small amounts of APP appear to be present in lipid rafts under physiological conditions (Bouillot et al., 1996; Parkin et al., 1999). Increasing membrane cholesterol levels may increase overall percentage of lipid rafts, which favors APP and BACE1 interaction and increases Aβ generation. Several studies support this idea. Firstly, imaging of fluorescently tagged APP and BACE1 demonstrates that cholesterol loading does not increase Aβ production through BACE1 catalytic activity but rather by altering the accessibility of BACE1 to its substrate APP in lipid rafts (Marquer et al., 2011). Secondly, APP and BACE1 copatch at the plasma membrane upon antibody cross-linking, which increases Aβ production in a cholesterol-dependent manner (Ehehalt et al., 2003). Lastly, inhibition of γ-secretase activity leads to an accumulation of APP-CTFs in lipid rafts (Vetrivel et al., 2004).

Niemann–Pick type C disease (NP-C) is a lysosomal lipid storage disorder, characterized by accumulation of cholesterol and sphingolipids within the endosomal–lysosomal system. The majority of NP-C cases are caused by functional loss of NPC1 protein activity, due to genetic mutation. Neuronal degeneration underlies neurological symptoms in NP-C patients, which include cerebellar ataxia, dysphagia, dysarthria, and dementia. Altered cholesterol distribution within subcellular compartments has been implicated in the aberrant trafficking and processing of APP similar to that observed in AD (Runz et al., 2002; Vanier and Millat, 2003; Jin et al., 2004; Walkley and Suzuki, 2004; Vance, 2006; Kodam et al., 2010; Kosicek et al., 2010; Malnar et al., 2010, 2012). In cell models of NP-C, cholesterol overload due to NPC1 deficiency leads to increased APP lipid raft localization and internalization from the plasma membrane to BACE1-rich endosomes, where amyloidogenic processing occurs (Kosicek et al., 2010; Malnar et al., 2010). This can be corrected by cholesterol depletion in cultured cells using lipid-deficient serum, lovastatin treatment, or methyl-β-cyclodextrin treatment (Malnar et al., 2012). The cholesterol-dependent change in APP trafficking and lipid raft localization parallels previous studies of APP’s response to changes in cellular copper levels (Hung et al., 2009; Acevedo et al., 2011). Furthermore, cholesterol-dependent APP trafficking and metabolism may explain some of the metal changes observed in NP-C tissue samples (Hung et al., 2014). Taken together, these evidences suggest a synergistic interaction between copper and cholesterol pathways in the regulation of APP metabolism that may contribute to AD pathogenesis.

Altered intracellular cholesterol metabolism can also affect APP processing. Cultured cells exposed to a cholesterol transport inhibitor, U18666A, accumulate cholesterol in late endosomes and lysosomes, and results in a dose-dependent decrease in Aβ production (Runz et al., 2002; Davis, 2008). However, the inhibitor also increases accumulation of γ-secretase, CTFβ, and Aβ-related peptides in vesicular organelles (Runz et al., 2002; Jin et al., 2004). From these studies, it can be inferred that cholesterol is able to influence APP processing through re-internalization of surface APP, as well as redistribution of APP and its processing enzymes within subcellular compartments.

### METAL MODULATION OF Aβ GENERATION, AGGREGATION, AND CELL TOXICITY

Aβ binds to zinc, copper, and iron to form various precipitous complexes, which are dependent on pH, buffer conditions, and initial peptide aggregation rate (Bush et al., 1994b; Huang et al., 1997; Garai et al., 2006; Tougu et al., 2008). Human Aβ binding of zinc, and both oxidized and reduced copper (Bush et al., 1994c; Atwood et al., 2000; Syme et al., 2004; Syme and Viles, 2006; Danielsson et al., 2007; Himes et al., 2008; Karr and Szalai, 2008; Shearer and Szalai, 2008; Hureau and Faller, 2009) is mediated by nitrogen ligands from histidine at positions 6, 13, and 14 together with an oxygen ligand (Curtain et al., 2001). Interestingly, rat and mouse have different amino acids at the metal ion coordination site, which could explain why these animals resist developing amyloid pathology compared to other mammals (Gaggelli et al., 2008). More details regarding the biophysical and biochemical binding of Aβ and the above mentioned metals have been reviewed (Faller and Hureau, 2009; Rozga et al., 2009).

Neurotoxic effects of Aβ depend on peptide aggregation, metal ion interaction, and generation of reactive oxygen species (ROS) with the subsequent formation of soluble covalently cross-linked oligomers. Both Cu:Aβ and Fe:Aβ complexes have been shown to exhibit cytotoxic effects (Schubert and Chevion, 1995; Liu et al., 2011; You et al., 2012), which can be rescued by chelation or competitive binding (Huang et al., 2004; Wu et al., 2008; Perrone et al., 2010). Interestingly, it has been shown that modifying copper binding histidine 6 or 13 to alanine induces significant cell toxicity in primary cortical cell cultures at levels similar to the wild-type peptide (Smith et al., 2010). However, modifying histidine 14 (a known ligand for copper and the cell plasma membrane), did not induce any measurable toxicity that correlates with the ability of the modified peptide to bind to cell membranes (Smith et al., 2010).

Under normal physiological conditions, non-toxic monomeric forms of Aβ are the predominant species (Haass et al., 1992; Vigo-Pelfrey et al., 1993; Shoji, 2002). However, pathological stimuli are thought to trigger complex conformational changes and assembly of Aβ peptides to form a heterogeneous mixture of oligomers and fibrils. This aggregation of Aβ is a critical event for neurotoxicity to occur. Soluble Aβ oligomers, and not fibrils, are currently considered the proximate neurotoxin in AD pathology (Dahlgren et al., 2002; Kayed et al., 2003; Cleary et al., 2005; Haass and Selkoe, 2007; Lesne et al., 2008; Roychaudhuri et al., 2009; Shankar and Walsh, 2009). However, as both Aβ oligomers and fibrils can interact synergistically with tau and cause mitochondrial function impairment in the P301L tau transgenic mouse model (Eckert et al., 2008), the distinction in all forms of neurotoxicity between Aβ species is not clear. Both copper and iron have been shown to modify Aβ and accelerate its aggregation *in vitro* (Mantyh et al., 1993; Atwood et al., 2000; Ali et al., 2005). Oxidation of the Aβ side-chain by copper leads to covalent oligomerization (Ciccotosto et al., 2004; Ali et al., 2005). Tyrosine at position 10 of Aβ is particularly susceptible to free radical attack. When complexed to Cu^2^^+^ or Fe^3^^+^ and in the presence of H_2_O_2_, Aβ forms dityrosine cross-linked oligomers, which are suggested to seed accelerated Aβ aggregation (Atwood et al., 1998, 2004; Barnham et al., 2004). Unlike zinc, copper mediates Aβ oligomer formation rather than amyloid fibrils, and thus Aβ:Cu oligomers are not recognized by the β-sheet marker, thioflavin T (Jiao and Yang, 2007; Tougu et al., 2009).

Investigations on metal-mediated modulation of Aβ have been carried out in APP transgenic models supplemented with either dietary copper or zinc. Administration of copper to APP23 mice, overexpressing human APP with the AD-related Swedish mutation, elevated copper levels in the brain compared to wild-type littermate controls, resulting in a lowering of soluble and insoluble Aβ (Bayer et al., 2003). Dietary zinc supplementation also reduced Aβ plaques in brains of Tg2576 (another transgenic mouse model carrying the Swedish-APP mutation) and TgCRND8 (a triple transgenic mouse model carrying APP with Swedish and Indiana mutations). However, AD-like spatial memory impairments are increased in the zinc-fed transgenic mice (Linkous et al., 2009). Conversely, decreased dietary zinc in a APP/PS1 transgenic mouse model of AD elevated plaque volume (Stoltenberg et al., 2007). Elevation of brain copper by crossing TgCRND8 with a transgenic mouse model deficient in the copper transporter, ATP7B, reduces plaque load as well as soluble and insoluble Aβ levels (Phinney et al., 2003). These evidences suggest that an intracellular shift in copper reduces Aβ aggregation.

Intracellular zinc export takes place through the zinc transporters (ZnT) protein family. Currently eight ZnTs are known, of which, ZnT-1 is the only member that exports zinc across the plasma membrane within the brain (Lovell et al., 2005). ZnT-3 transports zinc to glutamatergic vesicles in hippocampal granule, pyramidal, and interneuron cells (Cole et al., 1999; Linkous et al., 2008), ZnT-4 sequesters cytosolic zinc into acidic vesicles (Kelleher and Lonnerdal, 2002) and ZnT-6 sequesters zinc in the *trans*-Golgi network and vesicular compartments (Huang et al., 2002). The highest concentration of labile zinc is present in synaptic vesicles that are released during synaptic transmission of neocortical glutamatergic fibers. As mentioned, the activity of ZnT-3 is required for the passage and pooling of zinc within these pre-synaptic vesicles, making it available for an interaction with the Aβ that is predominantly located within the synapse. Crossing of ZnT-3 knockout mice with Tg2576 mice, reduces both cerebral plaque load (Lee et al., 2002) and amyloid angiopathy (Friedlich et al., 2004). This supports the theory that high concentrations of zinc in the synaptic cleft play a role in amyloid formation in AD.

Oxidative stress-induced damage of brain tissues is a major hallmark of AD. The redox chemistry involved in the production of toxic ROS from metal enriched Aβ complexes and general metal dyshomeostasis is implicated in this process. Binding of oxidized copper or iron to Aβ results in reduction of the metal valency state and subsequent production of H_2_O_2_ (Huang et al., 1999; Opazo et al., 2002; Tabner et al., 2002; Nelson and Alkon, 2005). This can be further exacerbated by the reaction of hydrogen peroxide with reduced metal to produce hydroxyl radicals through Fenton and Haber–Weiss reactions (Fenton, 1894; Haber and Weiss, 1934). Hydroxyl radicals are highly chemically reactive and contribute to generation of lipid peroxidation products, protein carbonyl modifications, and nucleic acid adducts such as 8-hydroxy guanosine, all of which feature strongly in AD neuropathology (Smith et al., 1996, 1997). Of note, evidence suggests that the biological reductants involved in Aβ redox cycling are most likely cholesterol and long chain fatty acids (Opazo et al., 2002; Barnham et al., 2004; Haeffner et al., 2005; Nelson and Alkon, 2005; Puglielli et al., 2005; Smith et al., 2006). This is consistent with experimental evidence demonstrating that toxicity associated with Aβ occurs on the plasma membrane (Ciccotosto et al., 2004). Additionally, the products of lipid oxidation such as oxysterols, 7β-hydroxycholesterol and 4-hydroxy-2-nonenal, which in turn increases Aβ cross-linking (Murray et al., 2005), are elevated in AD tissues and mouse models of the disease (Opazo et al., 2002; Haeffner et al., 2005; Nelson and Alkon, 2005; Puglielli et al., 2005; Smith et al., 2006).

### CHOLESTEROL MODULATION OF Aβ GENERATION, AGGREGATION, AND CELL TOXICITY

The majority of *in vivo* data provide support for an involvement of cholesterol in Aβ generation (Sparks et al., 1994; Bodovitz and Klein, 1996; Bouillot et al., 1996; Lee et al., 1998; Simons et al., 1998, 2001; Frears et al., 1999; Kojro et al., 2001; Wahrle et al., 2002; Ehehalt et al., 2003; Vetrivel et al., 2004; Kalvodova et al., 2005; Osenkowski et al., 2008). However, the impact of altering plasma cholesterol on brain Aβ generation remains unclear. Animal studies report no correlation (Parkin et al., 1999) or inverse correlation (Jin et al., 2004; Davis, 2008; Marquer et al., 2011) between dietary or peripheral cholesterol and Aβ. Several reasons can account for this disparity between studies, which include genetic background, the transgenes present, age, gender, and/or treatment conditions and environment. Another significant reason may be associated with the inherent selectivity of the BBB. Cholesterol in the brain is synthesized *de novo* and it is unclear to what extent peripheral or dietary cholesterol influences brain cholesterol levels due to limited BBB penetration. Moreover, most studies that examine the effects of high dietary cholesterol on Aβ levels fail to measure brain cholesterol levels in the same experimental settings. It is therefore uncertain if alteration of brain Aβ levels is due to cholesterol changes in the brain or some other indirect mechanism that is caused by the modulation of peripheral cholesterol. Effects of Aβ generation under *in vivo* paradigms and of cholesterol modulating genes on APP processing/Aβ generation have been reviewed recently in detail (Maulik et al., 2013). Results from these studies have shown strong evidence that modulating cholesterol synthesis (Crameri et al., 2006), intracellular trafficking (Burns et al., 2003; Bryleva et al., 2010; Kodam et al., 2010; Borbon and Erickson, 2011), uptake (Bales et al., 1997, 1999; Holtzman et al., 2000; Irizarry et al., 2000; Cao et al., 2006; Kim et al., 2009), and removal (Koldamova et al., 2005; Wahrle et al., 2005, 2008) causally influence APP processing and Aβ generation.

Cholesterol-rich lipid rafts may play a role in catalyzing the aggregation of Aβ to its neurotoxic oligomeric state. Aβ isolated from AD patients is associated with lipid rafts in a cholesterol-dependent manner and reducing cholesterol levels results in less aggregated Aβ peptides (Schneider et al., 2006). Cholesterol is likely to modulate Aβ aggregation through modifying raft composition. The ganglioside GM1, which is predominantly found in the central nervous system, can bind Aβ peptides in lipid rafts to form a complex that acts as an endogenous seed to promote amyloid oligomerization, aggregation, and subsequent fibril formation (Choo-Smith et al., 1997; Kakio et al., 2002; Kim et al., 2006; Okada et al., 2008; Matsuzaki et al., 2010). This has been shown to be a primary mediator of oxidative stress on plasma membrane (Zampagni et al., 2010). Some studies examining effects of cholesterol on Aβ toxicity *in vitro* provide evidence that decreasing cholesterol, sialic acid, and ganglioside synthesis is protective to PC12 cells, while increasing cholesterol levels lead to increased Aβ neurotoxicity (Wang et al., 2001; Lin et al., 2008). Interestingly, it has been observed that sustained ROS production is associated with Aβ toxicity when exogenous cholesterol is increased (Ferrera et al., 2008). Other studies disagree with these results. PC12 cells and cultured neurons with high cholesterol levels in the membrane are resistant to Aβ toxicity, while low cholesterol levels increase their susceptibility (Zhou and Richardson, 1996; Yip et al., 2001; Arispe and Doh, 2002; Sponne et al., 2004). These divergent results suggest a dynamic yet intricate correlation between cholesterol and Aβ peptide, such that cholesterol’s influence on physical properties of lipid rafts can modulate Aβ binding and aggregation to affect cell viability.

### METAL MODULATION OF Aβ DEGRADATION

The over-production of toxic Aβ is only one side of the equation that contributes to senile plaque production and AD pathology, with the other possible side, less frequently studied but equally important, involving a fault in the degradation and clearance regulatory pathways of Aβ (reviewed in Carson and Turner, 2002; Ling et al., 2003). Three proteases in the brain most frequently studied in Aβ degradation, are insulin-degrading enzyme (IDE), neprilysin (NEP), and plasmin. Of these three proteases, IDE and NEP are members of the zinc metallopeptidase family of proteins that have a zinc binding domain with common sequence homology that can be potentially altered with aberrant zinc metabolism (Vekrellis et al., 2000; Fan et al., 2009). Additionally, metal binding ligands of both enzymes are oxidatively modified in the AD brain by various ROS, such as hydroxyl radicals and products of ROS, such as 4-hydroxy-2-nonenal (Wang et al., 2003; Caccamo et al., 2005; Shinall et al., 2005). These data suggest that the generation of ROS, perhaps as a product of metal:Aβ redox cycling, may serve to inactivate proteases involved in Aβ degradation. Conversion of plasminogen to plasmin involves cleavage from either tissue-type (tPA) or urokinase-type plasminogen activator (Ledesma et al., 2003). Inhibition by tPA cleavage of plasminogen is again caused by increased redox cycling and production of ROS in the presence of copper/ascorbate (Lind et al., 1993). Plasmin itself may also be regulated by site-specific oxidation; in particular, modification of the histidine molecule that resides in its active site (Lind et al., 1993). Lastly, Aβ is a substrate for matrix metalloproteinase (MMP), and plasmin has been shown to activate MMP2 degradation of Aβ, a process that is inhibited in the presence of zinc but not copper (Crouch et al., 2009).

### CHOLESTEROL MODULATION OF Aβ DEGRADATION

A number of recent studies have shown that cholesterol may be involved in Aβ clearance by regulating Aβ degrading enzymes. After synthesis, IDE is transported via the secretory pathway to the cell membrane where it either remains or is secreted. Given that a subset of IDE is localized in lipid rafts (Bulloj et al., 2008), it is possible that cholesterol levels or distribution can regulate the transport and release of this protease to influence Aβ degradation. Similar to IDE, the mature form of NEP also associates with lipid rafts (Sato et al., 2012). Contradictorily, targeting NEP chimeric proteins to lipid rafts fails to efficiently degrade Aβ in this fraction (Hama et al., 2004). It is of note that plasmin is also a raft protein (Ledesma et al., 2003). Mice deficient in seladin-1, which is required for cholesterol synthesis, present disorganized rafts and impaired plasmin function (Crameri et al., 2006; Stefani and Liguri, 2009). These evidence supports the notion that cholesterol, possibly through raft maintenance, is required for plasmin degradation of Aβ.

### APOE ASSOCIATION WITH METAL AND CHOLESTEROL ON ITS ROLE OF Aβ CLEARANCE

There is strong evidence that ApoE plays a central, if not direct, role in the pathogenesis of AD. The human *APOE* gene exists as three polymorphic alleles (ε2, ε3, and ε4), and individuals possessing the ε4 allele are at highest risk of developing AD (Bales et al., 2009; Reiman et al., 2009; Castellano et al., 2011). ApoE is well known for its involvement in the transportation of cholesterol. Together with a multitude of other apolipoproteins, lipoprotein receptors, and lipid transporters, ApoE controls cholesterol homeostasis in the brain (brain cholesterol homeostasis reviewed in Hung et al., 2013). Studies in human and transgenic mice demonstrate an isoform dependent (ε4 > ε3 > ε2) accumulation of Aβ levels and amyloid plaque load.

Currently, there is no clear evidence that ApoE affects APP processing and Aβ production *in vitro* and *in vivo* (Biere et al., 1995; Cedazo-Minguez et al., 2001; Irizarry et al., 2004). However, ApoE appears to play an important role in Aβ clearance through several possible mechanisms. *In vitro* studies with neuronal cells have shown that lipidated ApoE binds to soluble Aβ in an isoform-dependent manner (ε2 > ε3 > ε4) and is internalized into various brain cells for degradation by receptor-mediated endocytosis (Beffert et al., 1998, 1999; Yang et al., 1999; Cole and Ard, 2000; Yamauchi et al., 2000, 2002). ApoE may also facilitate removal of Aβ from the brain through the BBB (Cirrito et al., 2005; Zlokovic, 2008). ApoE may be able to facilitate the cellular degradation of Aβ *in vitro*, however, the mechanism and whether it is isoform-specific still requires clarification (Crameri et al., 2006; Bryleva et al., 2010).

There are very limited studies investigating metal interaction with ApoE and its relationship with APP or Aβ. ApoE protein binds copper, iron, and zinc, suggesting that ApoE has the ability to sequester metals. This may underlie its isoform-dependent antioxidant activity (ε2 > ε3 > ε4; Miyata and Smith, 1996). Interestingly, ApoE4 contains a cysteine to arginine substitution at positions 112 and 158. Since cysteine is believed to be involved in transition metal binding, reduced affinity of ApoE4 to metal may therefore relate to diminished antioxidant effects of the ApoE4 allele (Moir et al., 1999). 

ATP binding cassette transporter A1 (ABCA1) is a cell surface membrane protein that promotes efflux of cellular cholesterol to acceptor molecules, including ApoE and ApoA1. The ApoE4 isoform has been found to reduce ABCA1-mediated cholesterol efflux in astrocytes and neurons *in vitro* (Michikawa et al., 2000; Gong et al., 2007). Studies involving ABCA1-deficient mice show poor lipidation with decreased levels of ApoE (70–80% reduction) and a concurrent increase in amyloid plaque burden (Wahrle et al., 2004, 2005; Hirsch-Reinshagen et al., 2005; Koldamova et al., 2005). Conversely, ABCA1 overexpression in mice result in increased lipidation and ApoE levels, and decreased amyloid plaque formation (Wahrle et al., 2008). Interestingly, both ApoE and ABCA1 can be modulated by transcription factor liver-X-receptors (LXRs), which may be a key regulator in brain lipid homeostasis. Indeed, deficiencies in LXRα and/or β augment AD pathology (Zelcer et al., 2007), whereas treating AD mice with LXR agonists, including Bexarotene, result in reduced amyloid plaque burden and improved cognitive function (Eckert et al., 2007; Riddell et al., 2007; Vanmierlo et al., 2011; Cramer et al., 2012).

## TWO SIDES OF THE SAME COIN: POSSIBLE CROSSTALK BETWEEN METALS AND CHOLESTEROL IN APP/Aβ METABOLISM

Independently, there are large bodies of research detailing the influence of metals or cholesterol on the development, progression, and pathogenesis of AD. However, there are limited studies on the relationship between metals and cholesterol in AD pathology. The review thus far gives an overview of the impact of either metals or cholesterol on the amyloidogenic and non-amyloidogenic processing pathways of APP. Interestingly, there are many overlaps between these two factors impacting various processes in both of these pathways. The review will now examine interactions between metals and cholesterol with APP and Aβ.

Epidemiological studies have shown that dietary intake of trans- and saturated fats lead to an unfavorable cholesterol profile in AD patients and may associate with cognitive decline (Morris et al., 2003, 2004). A follow-up study indicates that higher copper intake is associated with an accelerated rate of cognitive decline and that the copper-dependent acceleration is lost in individuals who did not consume a high fat diet (Morris et al., 2006). In animal studies, cholesterol-fed rabbits have exacerbated neurodegeneration following consumption of trace amounts of copper (Sparks and Schreurs, 2003). Lowering cholesterol levels in patients by atorvastatin (a cholesterol lowering drug which inhibits HMGCR) saw an increase in circulating ceruloplasmin levels (Sparks et al., 2005), a ferroxidase involved in iron homeostasis and due to its role as a plasma copper transporter, a surrogate marker of plasma copper status. Interestingly, like copper, increased dietary cholesterol leads to dysregulation of iron regulatory proteins in rabbits and also iron accumulation in Aβ plaques (Ghribi et al., 2006). An epidemiological study in a large cohort of adults found that abnormally high dietary cholesterol and iron intakes increase the risk of AD (Mainous et al., 2005).

Based on evidence from the studies reviewed in Sections “Metals and APP” and “Cholesterol and APP,” it may be inferred that both metals and cholesterol are able to modulate APP metabolism/expression through a process that remains to be elucidated. Conversely, APP itself can regulate metal and cholesterol homeostasis. Therefore, APP may be envisioned as a key regulator linking both metal and cholesterol homeostasis, whereby unregulated metal or cholesterol leads to a downstream effect on APP that may ultimately cause an erroneous outcome in collateral systems.

**FIGURE 2:**
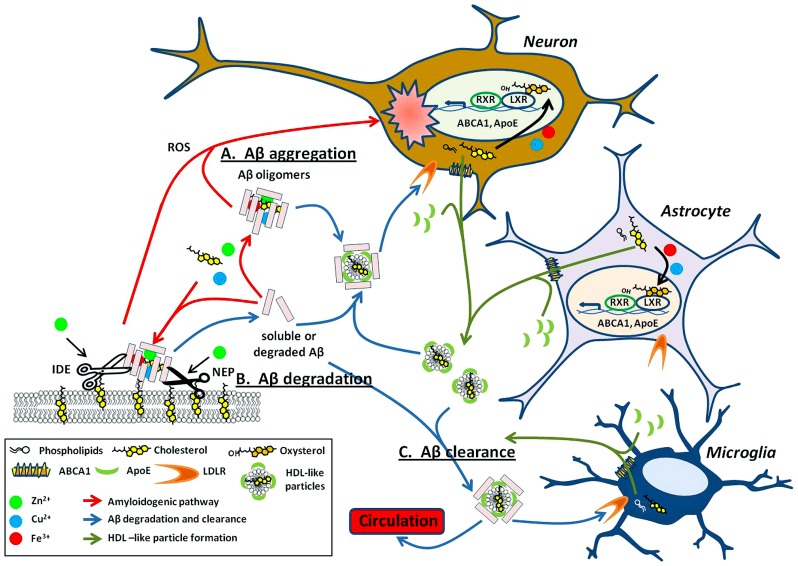
**Metals and cholesterol implicated in Aβ processing and neurotoxicity. (A)** Upon cleavage from APP, both metal and cholesterol bind to Aβ monomers promoting oligomerization of the peptide into multiple types of Aβ aggregates. These aggregates are either present within the extracellular space or bound to the surface of the plasma membrane (*red arrows*). Select Aβ aggregates are neurotoxic through multiple mechanisms such as their ability to generate reactive oxygen species (ROS) and may have implications in AD associated neuropathology. The ability of copper, iron, and cholesterol to promote redox cycling are acutely involved in the cytotoxicity caused by aggregated Aβ. **(B)** As with APP processing enzymes (**Figure 1B**), proteases that degrade Aβ, such as neprilysin (NEP) and insulin degrading enzyme (IDE) are dependent on metals for their catalytic activity. These proteases are also lipid raft associated and modulated by cholesterol levels in these domains. **(C)** Lipidated ApoE, produced mainly by astrocytes and microglia, binds soluble Aβ and facilitates its degradation through receptor-mediated endocytosis within neurons and microglial or clearance from the brain through the blood–brain barrier (BBB; *blue arrows*). Lipidated ApoE regulation is modulated by nuclear transcription factors LXRs. LXR heterodimerizes with RXR to transcriptionally regulate ABCA1 and ApoE. ABCA1 exports cellular cholesterol and phospholipids that in turn lipidate ApoE to form HDL-like particles (*green arrows*). These HDL-like particles are required for the clearance of Aβ. Intriguingly, the LXR agonist, oxysterols is elevated in the AD brain and may result from cholesterol oxidation by metals and 24S-hydroxylase. Evidence implies that the involvement of metal and cholesterol in the Aβ processing pathway is not just deleterious (as in **A**) but may also have importance in degradation and clearance of this potentially harmful peptide **(B,C)**.

One way in which copper, iron, and zinc can impact on the pathology of AD stems from their relative ease in switching oxidation states. This property makes it particularly useful for enzymatic reactions requiring electron transfer (Waldron et al., 2009). Metals can affect APP processing (**Figure 1B**) and Aβ degradation (**Figure 2**) by altering catalytic properties of secretases, which are metalloproteins (reviewed in Sections “Metal Modulation of APP Processing Enzymes” and “Metal Modulation of Aβ Degradation”). Cholesterol, on the other hand influences APP processing (**Figure 1B**) and Aβ degradation (**Figure 2**) through lipid raft association of substrates (APP and Aβ) and enzymes (APP processing secretases and Aβ degrading proteases; reviewed in sections “Cholesterol Modulation of APP Processing” and “Cholesterol Modulation of Aβ Degradation”). Cholesterol influences both the quantity and quality of the lipid raft domains. For example, cholesterol can modulate the order of raft components to provide the right environment for protein binding or function. In other words, metals modulate APP processing and Aβ degradation through the mechanistic action of the enzyme, whereas cholesterol does so through manipulation of the environment and presentation of the substrate and/or enzyme on the plasma membrane. Although this relationship between metals and cholesterol may not be mutually exclusive, a fault in either system may still lead to similar pathological outcomes in AD (**Figure 2**).

More recently, copper has been observed to directly influence the lipid raft protein, flotillin-2 (Hung et al., 2009). Flotillin-2 interacts with APP at the cell surface (Schneider et al., 2008). The endocytosis of APP to BACE1-rich endosomes, required for β-cleavage of APP, is sensitive to flotillin-2 depletion (Ehehalt et al., 2003; Schneider et al., 2008). Analogous to cholesterol depletion, elevated copper reduces flotillin-2 association with lipid rafts, thereby reducing endocytosis of APP and attenuating Aβ production (Hung et al., 2009).

Interestingly, both metals and cholesterol are able to catalyze the oligomeric aggregation of Aβ required for its cytotoxic effect (see “Metal Modulation of Aβ Generation, Aggregation, and Cell Toxicity” and “Cholesterol Modulation of Aβ Generation, Aggregation, and Cell Toxicity”; **Figure 2**). The mechanism of aggregated Aβ toxicity is still a matter of debate. However, elevation of ROS in both metal:Aβ or GM1:Aβ complexes suggests an involvement of metal as a mechanistic partner to redox cycle and generating harmful ROS products from both metal- and cholesterol-based aggregation of Aβ *in vitro*.

Oxysterols also play an important role in the regulation of cholesterol in the brain and the body. In the brain, oxysterols are produced by conversion of cholesterol to the oxidized species, 24OHC, by the enzyme 24S-hydroxylase. 24OHC represents one of the main forms of cholesterol that can be trafficked out of the brain to the circulatory system by its permeability across the BBB (Bjorkhem et al., 1998; Lund et al., 1999; Ehehalt et al., 2003; Lutjohann and von Bergmann, 2003; Schneider et al., 2008). Interestingly, APP has been shown recently to regulate 24S-hydroxylase levels (Pierrot et al., 2013). Oxysterols are agonists of LXRs, the latter of which form heterodimer complexes with retinoid x receptor (RXR) to transcriptionally regulate the production of a number of genes involved in the cholesterol regulatory pathway including ApoE, ABCA1, ABCG1, and SREBP1 (Bjorkhem, 2013). Through this pathway, oxysterols are able to regulate cholesterol efflux from cells via LXRs (**Figure 2**). In the progression of AD, levels of oxysterols are elevated, possibly due to effects of 24S-hydroxylase and non-enzymatic oxidation of cholesterol caused by elevated metal levels (Iuliano, 2011). This may be a way in which the brain is utilizing a feedback mechanism to clear excess cholesterol and Aβ peptides. Therefore, it is not surprising that elevated LXR-induced expression of ApoE4 (with defective Aβ and cholesterol clearance) compared to ApoE2, results in continued accumulation of neuritic plaques. The pathology of the disease continues to progress in a positive feedback loop of increased cholesterol, ROS, and Aβ generation (**Figure 2**).

## CONCLUSION

Metal and cholesterol are intrinsically linked to the pathogenesis of AD. Despite large bodies of research examining the abnormalities of metals and cholesterol in AD, the reciprocal influence of these two factors in the cause and progression of the disease remains to be elucidated. This review presents an overview of how metals and cholesterol independently impact upon the amyloidogenic and non-amyloidogenic processing of APP. It highlights the close and complex relationship between metals and cholesterol in the maintenance of normal brain physiology and the progression of AD pathology, with respect to interactions with AD-related proteins APP and Aβ. In the scheme of APP processing and Aβ metabolism, a disturbance to one homeostatic system may likely lead to a direct or indirect dysregulation of the other, although some of its molecular actions are mutually exclusive, the eventual deleterious outcome is the same. Continued research into metal or cholesterol influences on AD pathology must take careful consideration of the other factor, given that they are intrinsically linked. For example, the study of an influential effect metals has on ApoE should always take into account its lipidation status, which affects its conformation and activity. Conversely, the study of Aβ aggregation in lipid rafts should consider the role metals play in the generation of ROS products and subsequent cell toxicity. Further research is needed to investigate molecular mechanisms that link metals and cholesterol with various players involved in AD pathogenesis. This knowledge is critical for future design and implementation of effective therapeutic strategies to treat AD.

## Conflict of Interest Statement

The authors declare that the research was conducted in the absence of any commercial or financial relationships that could be construed as a potential conflict of interest.
